# Importance of Application Rates of Compost and Biochar on Soil Metal(Loid) Immobilization and Plant Growth

**DOI:** 10.3390/plants12112077

**Published:** 2023-05-23

**Authors:** Sayyeda Hira Hassan, Yassine Chafik, Marta Sena-Velez, Manhattan Lebrun, Gabriella Stefania Scippa, Sylvain Bourgerie, Dalila Trupiano, Domenico Morabito

**Affiliations:** 1 Department of Biosciences and Territory, University of Molise, 86090 Pesche, Italy; s.hassan@studenti.unimol.it (S.H.H.); scippa@unimol.it (G.S.S.); dalila.trupiano@unimol.it (D.T.); 2 Laboratoire de Biologie des Ligneux et des Grandes Cultures, Université d’Orléans, INRAE, USC 1328, LBLGC EA 1207, CEDEX 2, 45067 Orléans, France; yassine.chafik@etu.univ-orleans.fr (Y.C.); marta.sena-velez@univ-orleans.fr (M.S.-V.); sylvain.bourgerie@univ-orleans.fr (S.B.); 3 Laboratory for Improving Agricultural Production, Biotechnology and the Environment, Department of Biology, Faculty of Sciences, University of Mohammed First, BP717, Oujda 60000, Morocco; 4 Department of Environmental Geosciences, Faculty of Environmental Sciences, Czech University of Life Sciences Prague, Kamýcká 129, 16500 Prague, Czech Republic; lebrun@fzp.czu.cz

**Keywords:** soil amendments, application rates, mining technosol, *Arabidopsis thaliana*, plant growth

## Abstract

In this study, we investigated the effect of different rates of compost (20%, 40%, 60% *w*/*w*) in combination with biochar (0%, 2%, 6% *w*/*w*) on soil physiochemical properties and the mobility of arsenic (As) and lead (Pb), in addition to the ability of *Arabidopsis thaliana* (ecotype Columbia-0) to grow and accumulate metal(loid)s. All modalities improved pH and electrical conductivity, stabilized Pb and mobilized As, but only the mixture of 20% compost and 6% biochar improved plant growth. Plants in all modalities showed a significant reduction in root and shoot Pb concentrations compared to the non-amended technosol. In contrast, As shoot concentration was significantly lower for plants in all modalities (except with 20% compost only) compared to non-amended technosol. For root As, plants in all modalities showed a significant reduction except for the mixture of 20% compost and 6% biochar. Overall, our results indicate that the mixture of 20% compost with 6% biochar emerged as the optimum combination for improving plant growth and As uptake, making it the possible optimum combination for enhancing the efficiency of land reclamation strategies. These findings provide a foundation for further research on the long-term effects and potential applications of the compost-biochar combination in improving soil quality.

## 1. Introduction

Soil contamination with metal(loid)s has become a global serious concern due to increased anthropogenic activities, i.e., use of fertilizers in agriculture, as well as industrial, commercial or mining activities [1]. Mining activities lead to the production, leaching, and migration of a large amount of metal(loid)s in the surrounding area [2]. As a result, these soils are characterized by significantly elevated concentrations of metal(loid)s far exceeding background levels [3]. In addition to elevated metal(loid)s concentration, these soils often encounter extreme pH and nutrient deprivation, which makes it challenging to establish a vegetation cover [4]. In this context, it is necessary to improve soil metal(loid)s immobilization, and prevention of spreading to wider areas, prior to vegetation installation. Many studies have focused on removing or immobilizing metal(loid)s in soil with the help of several inorganic or organic amendments [5,6,7]. Among these organic amendments, biochar and compost have shown efficient results [8,9].

Biochar is obtained by the pyrolysis of biomass under limited oxygen conditions. Properties and utilization of biochar are mainly based on the type of feedstock and production conditions [10,11]. However, in general, biochar is characterized by an alkaline pH, a microporous structure, a high organic carbon content, a large specific surface area, the presence of surface functional groups, and a high cation exchange capacity [12]. These characteristics make biochar efficient in adsorbing and immobilizing metal(loid)s, reducing soil ecotoxicity and thus favoring plant development and fitness [13,14]. For instance, Simiele et al. [15] amended contaminated soil with 2.5% (*w*/*w*) of biochar, which induced a significant effect on soil properties and growth of *A. thaliana*.

However, biochar’s available nutrients are sometimes low and need to be added through other amendments, rich in organic matter and available nutrients, such as compost [16]. Compost is rich in humus substances, plant nutrients, and other trace elements, which are helpful in improving soil fertility and plant growth [16]. Besides this, compost can potentially sorb metal(loid)s [17,18]. Biochar and compost could have mutual beneficial effects [19,20]; compost brings nutrients that are absent (or not available) from biochar while biochar stabilizes those nutrients and increase the period during which compost will be beneficial. For instance, a higher increase in pH has been observed after the combined application of both biochar and compost rather than when they were applied individually [21,22]. Thus, to boost the efficiency of two soil-restoring agents, compost and biochar can be combined thoroughly in order to enhance each other’s properties and thus effects. Many studies have demonstrated their combined efficacy in improving soil physicochemical properties, immobilizing metal(loid)s, and finally allowing plant growth. Sigua et al. [23] observed that the combination of compost and biochar, both applied at different rates of 0, 2.5, and 5.0% (*w*/*w*), enhanced the phytostabilization of Zn and Cd and improved the biomass of corn (*Zea mays*) growing on mine soil. A combined mixture of compost (25 g) and biochar (25 g) reduced the mobility and bioavailability of metal(loid)s in wetland soil affected by mining activities and industrial wastewater [24]. Contrary to this, Seehausen et al. [25] reported a neutral or antagonistic effect of the combined application of biochar and compost on the growth of *Abutilon theophrasti* (annual plant) and *Salix purpurea* (perennial plant). However, these studies are limited to using a unique ratio of compost and biochar. To the best of our knowledge, no study has been conducted to investigate and compare the impact of combination of compost and biochar at multiple application rates on mining technosol. It is worth noting, however, that the synergistic effects between biochar and compost may depend on the ratio between them. Thus, this study was conducted to compare different rates of compost and biochar in combination to evaluate their efficacy and to determine the optimum mixture to improve the quality of a mining technosol allowing plant growth. Compost was used at different rates (20%, 40%, 60%, *w*/*w*) alone or in combination with 0%, 2%, or 6% (*w*/*w*) biochar. Amendment mixtures were used subsequently in a pot trial with contaminated technosol from the mining district of Pontgibaud. The intensive mining activity contaminated the area with high concentrations of lead (Pb) (11,453.63 ± 0.18 mg·kg^−1^) and arsenic (As) (539.06 ± 0.01 mg·kg^−1^) [26], and left the tailings very acidic, with a sandy texture. Both Pb and As are characterized as the most prevalent potentially toxic elemental contaminants [27] due to their increased environmental mobility, adversity, and persistency, possessing toxic impacts on living beings and natural resources [28]. Physiochemical properties and metal(loid)s concentration in soil pore water of different soil combinations were measured on different days. Moreover, *Arabidopsis thaliana* (ecotype Columbia-0) was used to measure plant growth and metal(loid)s accumulation in plant organs under these modalities.

## 2. Results

### 2.1. Evaluation of the Soil Pore Water (SPW) pH and Electrical Conductivity (EC)

**Figure 1:**
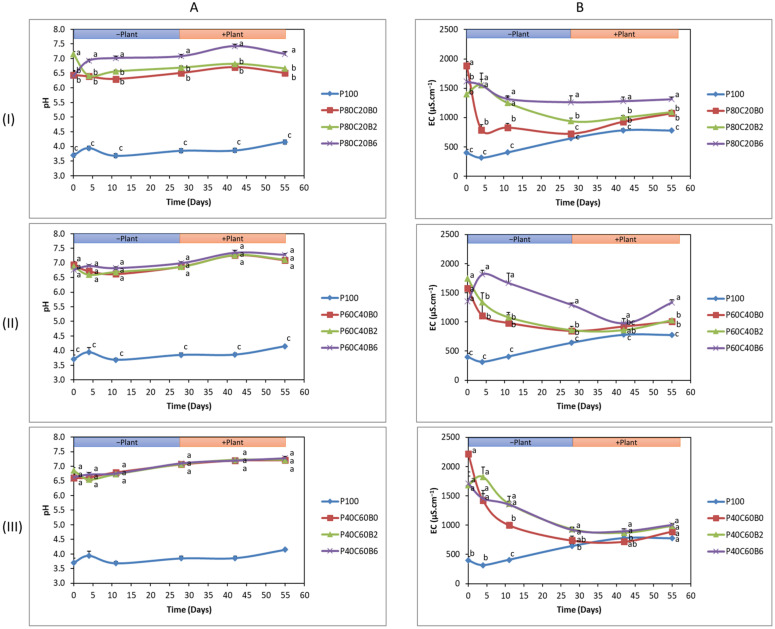
Analysis of pH (**A**) and electrical conductivity (EC) (**B**) in soil pore water (SPW under different modalities at different time points: before planting at Day 0 (D0), D4, D11, D28, and after planting at D42 and D55. Different modalities are as follows: Pontgibaud technosol (P100), Pontgibaud (P) amended with 20% compost without/with 2% and 6% biochar (P80C20B0; P80C20B2; P80C20B6), P amended with 40% compost without/with 2% and 6% biochar (P60C40B0; P60C40B2; P60C40B6), and P amended with 60% compost without/with 2% and 6% biochar (P40C60B0; P40C60B2; P40C60B6). Modalities were compared group-wise to evaluate the significance, and letters indicate significant differences at *p* < 0.05 among mean observations on same day between treatments.

From D0 until the end of the experiment, the pH of non-amended Pontgibaud technosol (P100) SPW remained acidic, ranging from 3.7 ± 0.2 to 4.2 ± 0.07 (Figure 1A). Regardless of the concentration, the addition of compost alone or in combination with biochar (2% and 6%) to technosol increased SPW pH and EC significantly (*p* < 0.05). The impact of modifications on raising pH differed significantly within group 1 (P80C20) (Figure 1A(I)) as the treatment P80C20B6 had a significantly higher pH (7.16 ± 0.07) than the control and other treatments at the end of the experiment (D55), whereas the treatments P80C20B2 and P80C20B0 had lower maximum pH values (6.67 and 6.51, respectively), with no significant differences between them. However, different results were observed in group 2 (P60C40) and 3 (P40C60), where the addition of 2% or 6% biochar did not cause a rise in pH in comparison with 0% biochar (without biochar), which points towards the main effect of higher dose of compost towards increased pH. In group 2, the maximum pH (7.28 ± 0.08) was observed for P60C40B6 without any significant difference with P60C40B2 (pH 7.11) and P60C40B0 (pH 7.10) (Figure 1A(II)) at D55. The same scenario was seen for pH in group 3, with no significant difference between the treatments, and the highest pH (7.28 ± 0.07) was recorded for P40C60B6 followed by P40C60B2 (7.22) and P40C60B0 (7.21) (Figure 1A(III)).

The EC of technosol SPW ranged from 396 ± 62 μS·cm^−1^ to 776 ± 67 μS·cm^−1^ (Figure 1B). The addition of compost alone or in combination with biochar (2% and 6%) to technosol significantly enhanced the EC. A sigmoidal-like decline was observed for the mixture of Pontgibaud and compost without biochar (B0%) in all three groups from Day 0 to Day 4, but nevertheless, the treatment P80C20B0 displayed 2.5–5 times higher EC values as compared to P100, whereas P60C40B0 and P40C60B0 showed 3.5–5.7 and 4.5–5.7-times higher EC values, respectively, in contrast to control P100 (Figure 1B). In group 1, P80C20B6 achieved the highest EC value (1312 ± 63 μS·cm^−1^) at Day55 with a significant difference with other treatments P80C20B0 (1072 ± 57 μS·cm^−1^) and P80C20B2 (1094 ± 27 μS·cm^−1^) (Figure 1B(I)). In group 2, P60C40B6 showed a fluctuating trend of EC, increasing from Day 0 (1350 μS·cm^−1^) to Day 4 (1818 μS·cm^−1^) and then decreasing to 976 μS·cm^−1^ on Day 42 and showed the highest EC value (1334 μS·cm^−1^) at Day 55 in comparison with other treatments (Figure 1B(II)). In group 3, no significant difference was found between treatments and the control at Day 55, with the highest EC achieved in the case of P40C60B6 (1002 ± 31 μS·cm^−1^) followed by P40C60B2 (985 μS·cm^−1^) > P40C60B0 (890 μS·cm^−1^) > P100 (776 μS·cm^−1^) (Figure 1B(III)).

### 2.2. As and Pb Concentration in SPW

**Figure 2:**
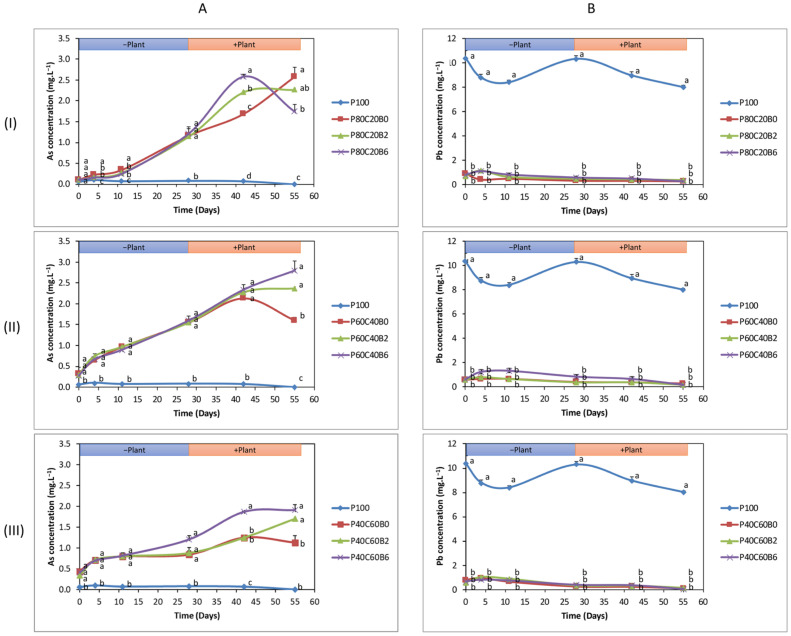
Analysis of As (**A**) and Pb (**B**) concentration (mg·L^−1^) in soil pore water (SPW) under different modalities at different time points: before planting at Day 0 (D0), D4, D11, D28, and after planting at D42 and D55. Different modalities are as follows: Pontgibaud technosol (P100), Pontgibaud (P) amended with 20% compost without/with 2% and 6% biochar (P80C20B0; P80C20B2; P80C20B6), P amended with 40% compost without/with 2% and 6% biochar (P60C40B0; P60C40B2; P60C40B6), and P amended with 60% compost without/with 2% and 6% biochar (P40C60B0; P40C60B2; P40C60B6). Modalities were compared group-wise to evaluate the significance, and letters indicate the significant differences at *p* < 0.05 among mean observations on the same day between treatments.

In comparison with control (P100), all modalities induced a significant increase in As concentration in SPW from the beginning to the end of the experiment (excluding modalities in group 1 at Day 0) (Figure 2). The treatments in each group followed the same increasing trend towards As concentration in SPW until Day 42; the highest As concentration was found in the case of P80C20B6 (2.58 ± 0.06 mg·L^−1^) in group 1 (Figure 2A(I)), P60C40B6 (2.34 ± 0.13 mg·L^−1^) in group 2 (Figure 2A(II)), and P40C60B6 (1.87 ± 0.02 mg·L^−1^) in group 3 (Figure 2A(III)). However, this scenario was different for group 1 on Day 55, where P80C20B6 showed a sharp decline in As concentration (1.74 ± 0.17 mg·L^−1^), and the highest As concentration (2.58 ± 0.23 mg·L^−1^) was seen in the case of P80C20B0 in group 1. The opposite scenario was observed for Pb concentration; where the addition of compost without/with 2% or 6% biochar to technosol significantly lowered the Pb SPW concentration compared to the control (P100) (Figure 2B). However, no significant difference could be seen in the effectiveness of different modalities in each group. The lowest Pb concentration in SPW was achieved by P80C20B6 (0.29 ± 0.03 mg·L^−1^) in group 1 (Figure 2A(I)), P60C40B6 (0.18 ± 0.06 mg·L^−1^) in group 2 (Figure 2A(II)) and P40C60B6 (0.06 ± 0.03 mg·L^−1^) in group 3 (Figure 2A(III)).

### 2.3. Plant Dry Weight and Metal(Loid) Concentration

**Figure 3:**
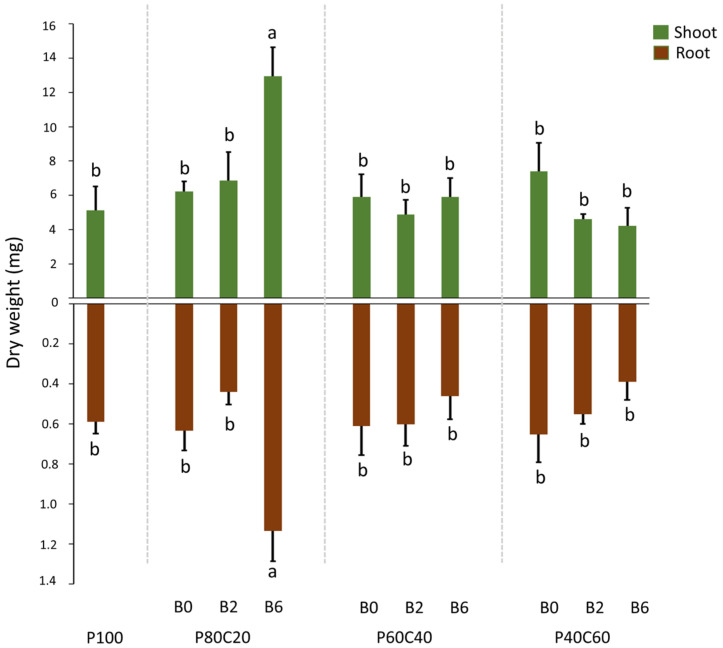
Dry biomass (mg) of root (brown) and shoot (green) of *A. thaliana* at 55 days under different modalities: Pontgibaud technosol (P100), Pontgibaud (P) amended with 20% compost without/with 2% and 6% biochar (P80C20B0; P80C20B2; P80C20B6), P amended with 40% compost without/with 2% and 6% biochar (P60C40B0; P60C40B2; P60C40B6), and P amended with 60% compost without/with 2% and 6% biochar (P40C60B0; P40C60B2; P40C60B6) (mean ± SE with *n* = 4). Letters on bar graphs indicate a significant difference (*p* < 0.05).

Measurements of dry biomass of shoot and root of *A. thaliana* (Figure 3) showed a strong effect of plant development for P80C20B6 with the highest root (1.13 ± 0.15 mg) and shoot (12.95 ± 0.15 mg) biomass with a significant difference from other treatments and control for both root and shoot. Other modalities showed no significant difference from the control.

**Figure 4:**
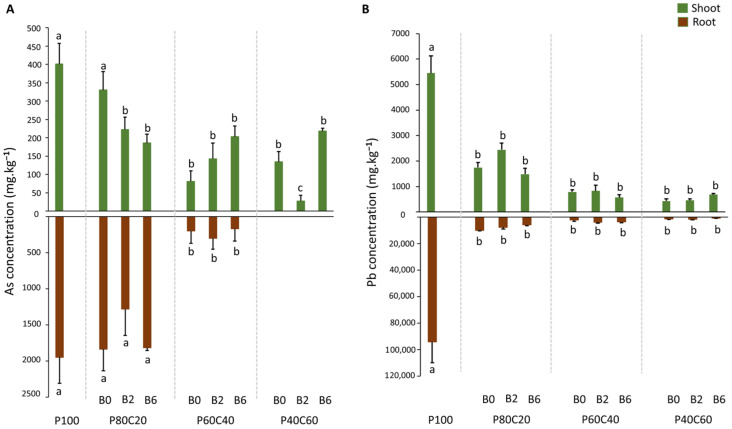
Arsenic (As) (**A**) and lead (Pb) (**B**) concentration (mg·kg^−1^) in root (brown) and shoot (green) of *A. thaliana* at 55 days under different modalities: Pontgibaud technosol (P100), Pontgibaud (P) amended with 20% compost without/with 2% and 6% biochar (P80C20B0; P80C20B2; P80C20B6), P amended with 40% compost without/with 2% and 6% biochar (P60C40B0; P60C40B2; P60C40B6), and P amended with 60% compost without/with 2% and 6% biochar (P40C60B0; P40C60B2; P40C60B6) (mean ± SE *n* = 4). Letters on bar graphs indicate significant difference (*p* < 0.05).

In all cases, metal(loid) concentration was higher in the roots than in the shoot of *A. thaliana* (except for the treatment P40C60 with 0%, 2% or 6% biochar in which no As was detected in roots) (Figure 4). As concentration in the roots of *A. thaliana* ranged from 0–1835 mg kg^−1^ (Figure 4A, Brown bars) and in the shoot between 81–330 mg kg^−1^. Plants from group 1 did not show any significant change in As concentration in the root in comparison with P100, independently of biochar percentage. However, plants in group 2 showed a significantly lower As concentration in roots, between 6–12 times lower than the control (P100). It can be noted that no As was found in roots of plants grown in treatments in group 3 (P40C60), whatever the dose of biochar added. On the other hand, As shoot concentration was significantly lower for plants in all modalities (except P80C20B0) compared to the P100 (Figure 4A, Green bars). For Pb, the range varied from 727 to 9829 mg kg^−1^ in the root and 425 to 2439 mg kg^−1^ for the shoot (Figure 4B). All plants showed a significantly lowered Pb concentration in the root and shoots compared to the control P100, and unlike As concentration, dosage of compost and/or biochar had no influence (Figure 4B).

**Figure 5:**
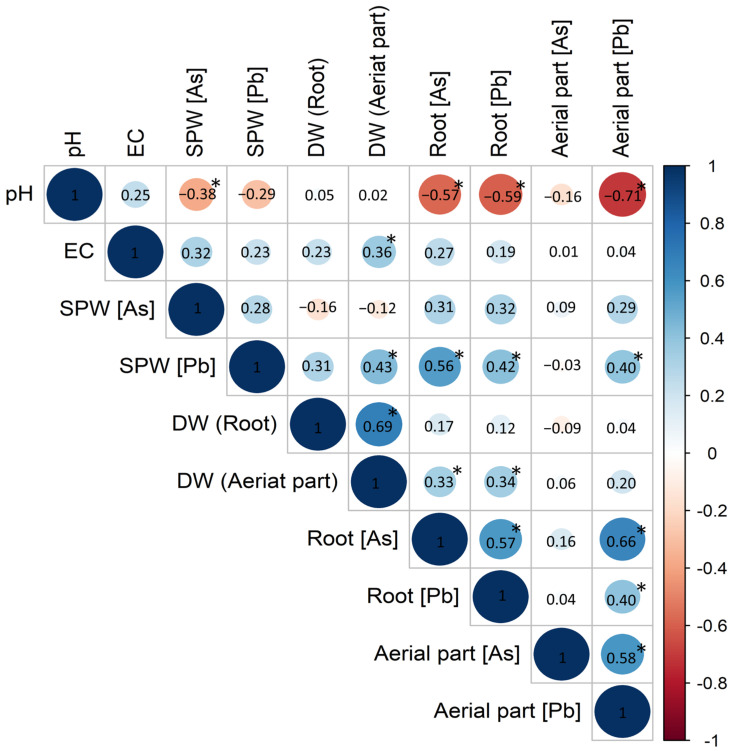
Correlations between pH, electrical conductivity (EC), metal(loid)s in soil pore water (SPW), in organs’ dry weight (DW), and in *Arabidopsis thaliana* organs. Blue color represents positive correlation and red color shows negative correlation. * Correlation is significant at the 0.05 level.

Finally, Pearson correlation was calculated to evaluate the association between different parameters (Figure 5). We found a significant negative correlation of pH with As concentration in SPW (−0.38) and root (−0.57), and with Pb concentration in root (−0.59) and aerial part (−0.71). A significant positive correlation was observed for SPW Pb concentration with dry weight (DW) of aerial part (0.43), root As level (0.56), Pb concentration in root (0.42) and aerial part (0.40). A weak positive correlation was seen for the DW of the aerial part with root As (0.33) and Pb concentration (0.34). Other positively correlated pairs were EC—aerial part DW (0.36), root DW—aerial part DW (0.69), root Pb—aerial part Pb (0.40), root As—root Pb (0.57) and aerial part As—aerial part Pb (0.58).

## 3. Discussion

The unamended Pontgibaud technosol presented an extreme acidic pH and the addition of compost without/with 2% or 6% biochar improved the technosol pH. In group 1, the addition of 6% biochar to technosol was more efficient in increasing SPW pH followed by 2% biochar and without biochar. Similarly, Lomaglio et al. [29] showed that the addition of high rate of biochar (5%) increased SPW pH more than the lower rate (2%). Lebrun et al. [30] also showed a higher increase in SPW pH of Pontgibaud technosol after adding the combination mixture of biochar (5% *w*/*w*) and compost (5% *w*/*w*) than adding compost alone (5% *w*/*w*). This increase in pH with the addition of or an increase in biochar amount can be attributed to the alkaline nature of biochar inducing a liming effect [30,31] and the release of base cations to be used by proton consumption reactions in the soil [31]. However, in group 2 and 3, the addition of 2% or 6% biochar to P60C40 and P40C60 did not show any significant difference in increasing SPW pH, when comparing the modalities, which shows the possible increase in soil buffering capacity by compost, which implies it can withstand pH fluctuations, necessitating the use of more biochar to raise pH.

The SPW EC of P100 was very low and all modalities with compost alone or in combination with 2% and 6% biochar increased SPW EC as compared to the P100. In groups 1 and 2, the best results were achieved by the addition of 6% biochar, while adding 2% biochar or without biochar did not have any effect. It suggests that ahigher amount of biochar should be used to increase SPW EC, which could be related to the enhancement of nutrient leaching into the soil solution [29]. Lomaglio et al. [29] also reported a more important increase in SPW EC after the addition of 5% biochar than 2% hardwood biochar to the mining technosol. Moreover, the mixture of technosol with compost alone in all groups (P80C20B0, P60C40B0, P40C60B0) showed a sharp decline in SPW EC from D0 to D4. These results can be due to the exchange between metal ions on biochar particles and metal(loid)s present in SPW, in comparison to the compost with the bad ion exchange [26,32]. Moreover, modalities in group 3 (P40C60) did not show any significant difference in SPW EC with the comparison with P100, irrespective of the presence/absence of biochar. This highlights the possible link of large amounts of compost having low ion exchange with SPW.

A large increase in SPW As concentration in contaminated soil was observed following the application of all modalities. In group 1, the highest SPW As concentration was observed for P80C20B6 followed by P80C20B2 and P80C20B0 until day 42, after which the scenario changed as SPW As showed a sharp decline under the treatment P80C20B6 (still higher than control) and the highest SPW As concentration was observed for modality with compost alone (P80C20B0) and adding 2% or 6% biochar to P80C20 decreased the SPW As concentration in comparison. This suggests that initially the combination of 20% compost and 6% biochar may have provided the most favorable conditions for the least arsenic release from the SPW. However, with the passage of time and the progression of decomposition and sorption processes, the scenario changed and the SPW As concentration declined under this treatment combination. This may be related to the addition of biochar as reported by Lebrun et al. [30], where the addition of 5% biochar to Pontgibaud technosol decreased SPW As concentration in both vegetated and non-vegetated pots, compared to the application of both biochar and compost or compost alone. On the other side, in group 2 and 3, the maximum increase in SPW As was found after the addition of 6% biochar to P60C40 and P40C60, respectively, followed by 2% biochar and without biochar. This contradiction in groups could also be related to the high amount of compost used in group 2 (40%) and 3 (60%), as it is widely known that compost discharges immense quantities of organic carbon content to the soil solution which then competes with As for sorption sites, resulting in an increase in As mobility [33,34]. These findings are noteworthy as in the case of modalities in group 2 and 3, a lower soil mass correlates with reduced levels of As. However, despite this decrease, there is still an Increase in As mobility, providing a clear indication of the amendment’s impact on As. Contrary to As, SPW Pb concentration was effectively decreased, at similar levels, following the application of all amendment mixtures. This strongly suggests the main role of compost in alleviating SPW Pb concentration. Lebrun et al. [30] also showed the ability of compost alone to cause the highest and most significant decrease (99%) in Pontgibaud SPW Pb in comparison with the other modalities in non-vegetated pots. This ability of compost can be attributed to its capacity to sorb metal(loid)s to its maximum level of organic matter as described by Karami et al. [35]. Huang et al. [17] also described the biosorbent role of compost towards metal(loid)s due to the presence of humic substances containing several organic functional groups. Other studies mainly attributed biochar for its role in reducing Pb bioavailability and mobility by sorbing Pb on the surface [36,37]. Other possible reasons behind the lack of significant difference between modalities, even after adding biochar, could be due to the insufficient sorption sites or clogged micropores on biochar surface due to the compost-derived materials [38]. Moreover, multiple studies described the mechanisms by which metal(loid)s are immobilized by amendments such as (i) metal adsorption through interactions with oxygenated functional groups present on the surface of biochar, (ii) presence of humic acid contents in the compost, and (iii) precipitation with carbonates and phosphates contained by biochar [29,30,39].

In addition to this, other studies also linked the addition of biochar with increased As in SPW as Zheng et al. [40] reported a subsequent increase of SPW As concentration after applying 5% *w*/*w* biochar (produced from parts of *Oryza sativa*) on mine soil. Beesley et al. [41] found an increase of around ninefold in SPW As concentration after 1 week of 30% *v*/*v* biochar (sourced from orchard prune residues) application, and correlated this to the increase in pH by biochar addition. An increase in pH causes a reduction in positively charged species on the mineral matrix, which further reduces the sorption capacity of negatively charged oxy-anions of As [42]. Contrary to this, Norini et al. [43] did not find any link between increased pH by the addition of hardwood sourced biochar (2% or 5%) and increased As in SPW. Rather, we found a weak negative but significant correlation (r = −0.38, *p* < 0.05) between pH and SPW As (Figure 5), which also suggest no involvement of increased pH in increasing SPW As in this case. Other factors behind the SPW As increase by adding biochar could be phosphate (P), as a recent study conducted by Glaser and Lehr [44] using meta-analysis reported that the application of biochar significantly enhances phosphorous availability in acid and neutral soils by factors of 5.1 and 2.4, respectively. Being chemically analogous to arsenate (As(V)), P strongly competes with As for sorption sites and thus facilitates As into the solution. In addition to P, dissolved organic carbon (DOC) increase by biochar could also compete with As for sorption sites, facilitating more As mobility [19,45]. Moreover, soluble complexes of As-DOC can be formed, increasing the mobility of As [46]. Thus, we can relate increased SPW As with combinational factors including increased amount of compost, and possible enhanced availability of P and DOC in soil solution. In modalities with application of 20% compost combined with 6% biochar, the dry weight for both root and shoots was significantly higher compared with the control, while plants in other modalities (P80C20B0, P80C20B2) of the same groups did not show any change in dry weight with respect to the control. This suggests that the higher amount of biochar should be used to improve plant growth in terms of dry weight, as biochar application may increase the nutrient retention in soil by increasing pH [47]. Furthermore, plants in other modalities of group 2 or 3 did not show any significant increase in dry weight as compared to the control, irrespective of adding compost or biochar. This might have resulted due to the combination of large amounts of compost with biochar, resulting in an oversupply of micronutrients, which could be toxic to the plants [25,48,49]. Seehausen et al. [25] found an antagonistic or neutral interactive effect of biochar and compost on *Abutilon theophrasti* growth and physiological functions; specifically, plant height and maximum leaf area were most impaired by the combination. Although many studies highlighted the combination of compost and biochar as a promising strategy to improve plant growth, which is thought to be mediated with positive synergistic effect of compost-biochar mixtures by enhanced sorption of nutrients, microbial colonization, degradation of noxious substances and sorption of dissolved organic carbon [50,51,52,53], there is still a lack of explicit studies to analyze synergistic effects with only few studies that used a factorial experimental design, also mentioned by Seehausen et al. [25].

Both As and Pb were more concentrated in plant roots than shoots for all modalities, which reflects the common defense strategy response including metal(loid) binding to the cell wall in response to avoid metal toxicity in plants [54]. In addition, the retention of high level of As and Pb in roots of *A. thaliana* describes its well-known ability to phytostabilize the metal(loid)s as reported by Simiele et al. [15] where higher metal(loid) concentrations were found in the roots of *A. thaliana* growing in Pontgibaud technosol alone or in combination with biochar and bacterial addition into soil. The same trend was seen for *Oxalis pes-caprae L.* growing in Pontgibaud technosol [26]. Vamerali et al. [55] also reported the higher concentration of metal(loid)s in roots of several species of *Populus* and *Salix* in metal-contaminated pyrite wastes. This aspect could be beneficial for the plants in terms of that root containment could prevent metal toxicity [56]. However, roots accumulated more Pb than As, which may be due to the fact that Pb has more affinity for root cells and a limited quantity is translocated to shoots [57,58]. Plants in all modalities showed a significant reduction in root and shoot Pb concentration compared to the control (P100), pointing towards less uptake and translocation of Pb either in the absence or the presence (2%, 6%) of biochar. As plants are able to uptake metal(loid)s dissolved in soil solution, or weakly bound to soil particles, so immobilizing metal(loid)s in solid phase by organic amendments can restrict their uptake by plants. We also observed a negative correlation of SPW Pb (r = 0.42, *p* < 0.05) with root Pb concentration. Thus, it can be assumed that it is mainly compost that enhanced the phytostabilization of Pb by restricting its bioavailability probably through forming organo-metal complex [32] and by decreasing SPW Pb mobility as discussed above. On the other side, roots of plants grown in Pontgibaud technosol amended with 20% compost alone and with 2/6% biochar (P80C20B0; P80C20B2; P80C20B6) showed highest root As concentration compared to other modalities, which can be related to the high dry weight of the plant roots in this group. Indeed, As concentration in roots of plants in these modalities did not show any difference from P100. This insignificance can be related to low SPW pH of P100, which is one of the main factors controlling the availability of metal(loid)s in soil. In this study, a strong negative correlation was found between SPW pH and As concentration in root, which is in accordance with Bhattacharya et al. [59] where a strong negative correlation was seen between soil pH and As concentration in all parts of rice plant. Modalities in group 2 significantly lowered the root As uptake as compared to the control and without any significant difference among them. Furthermore, no As was detected in roots growing on modalities of group 3, which shows the link between high amount of compost and less bioavailability of As. Moreover, compared to control, plants in all modalities displayed lower shoot As concentration (except for P80C20B0) which suggests the ability of these combinations to reduce As bioavailability and uptake by plants. While all modalities showed a decrease in shoot As concentration compared to the control, the plants in the P40C60B2 modality exhibited the most significant reduction among all treatments. This observation could be attributed to the higher proportion of compost (60%) combined with biochar, which may have further decreased As bioavailability in the soil, resulting in more limited uptake and translocation to the aerial part. However, due to the limited information, further investigations are suggested to confirm these hypotheses and understand the mechanisms involved in the differences among treatments.

## 4. Materials and Methods

### 4.1. Soil and Amendments

Soil samples were taken from a former mine settling pond (between 0–20 cm depth) located in the area “Roure-les-Rosiers” (GPS coordinates: 45°49′59″ North and 2°51′04″ East). The physicochemical properties of Pontgibaud technosol were evaluated in previous studies [60,61,62] and presented in Supplementary Table S1. Biochar (La Carbonerie, Crissey, France) was produced from the slow pyrolysis, at 500 °C, from a mixture of dry woody biomass containing beech, oak, and charm chips and wafers, followed by a sieving to obtain a particle size between 0.5 and 1 mm. The main physico-chemical properties of the biochar were studied by study by Lebrun et al. [63] and are also presented in Supplementary Table S1. The compost used in this study was commercial product composed of peat moss with a particle size range of 0–5 mm and having an electrical conductivity of 35 mS·m^−1^, 80% water retention capacity, and pH 6 (Klasmann-Deilmann, Saint-Louis-du-Rhône: France).

### 4.2. Experimental Design

Based on our prior research (not published), we found that there was no discernible difference in the effect of 2% and 4% biochar combined with compost. Thus, we chose a larger application rate of 6% biochar to be compared with 0% and 2% biochar. For compost, we chose application rates of 20%, 40%, and 60% since it is crucial to make sure the plants can develop and flourish in the testing environment [64] and in some circumstances, utilizing more compost may be necessary to give the plants the right nutrition and support. Consequently, nine treatments were used in the experiment, each with varying amounts of compost (20%, 40%, and 60%, *w*/*w*) and biochar (0%, 2%, and 6% *w*/*w* in relation to the total weight of the soil-compost mixture), along with Pontgibaud technosol and the treatment nomenclature is as follows: group 1 includes Pontgibaud 80% and compost 20% with 0% biochar (P80C20B0), 2% biochars (P80C20B2) or 6% biochar (P80C20B6); group 2 consists of Pontgibaud 60% and compost 40% with 0% biochar (P60C40B0), 2% or 6% biochar (P60C40B2 and P60C40B6, respectively). Group 3 contains Pontgibaud 40% and compost 60% mixture formulated with 0% biochar (P40C60B0), 2% or 6% biochar (P40C60B2 and P40C60B6, respectively). Each group was compared with 100% Pontgibaud technosol (P100) without any amendment as a control (Table 1). Four 400 mL plastic pots (8.7 × 11.3 cm) were prepared for each treatment.

### 4.3. Growth Conditions

The experiment lasted for 55 days, during which the first 28 days corresponded to the mixture equilibration and by day 28, three uniform 14-day-old rooted seedlings (grown in compost) of *A. thaliana* (ecotype Columbia-0) were transferred to each pot. Then, the plants were allowed to grow for 27 days in a growth chamber under 16-h light/8-h dark photoperiod and a temperature of 22 ± 2 °C, with watering when needed.

### 4.4. SPW Collection and Analysis

To investigate the effect of different amendments, the SPW was collected at Day 0 (D0), D4, D11, D28, D42, and D55 to evaluate pH and electrical conductivity (EC) and to determine the soil’s total As and Pb concentration. To collect SPW, soil moisture samplers (Rhizon^®^; Rhizosphere Research Product, Wageningen, The Netherlands) were placed at 40° in each pot at the beginning of the experiment and kept in the pot for the entire experiment duration. SPW samples were used to measure pH and EC using a combo pH and EC multimeter (Metler-Toledo, Seven excellence). Further, SPW samples were acidified with HNO_3_ prior to ICP-AES (inductively coupled plasma-atomic emission spectroscopy; ULTIMA2, HORIBA, Labcompare, San Francisco, CA, USA, with a detection limit of below 100 ppb) analysis to determine total dissolved As and Pb concentrations.

### 4.5. Plant Dry Weight and Metal(Loid)s Concentration

Plants were harvested after 27 days of growth (experiment day 55) on the different substrates. Roots and shoots were collected separately, washed carefully with de-ionized water to avoid loss of biomass and remove adhered soil particles. Subsequently, plant samples were dried at 60 °C for 36 h to determine dry weight. Roots or shoots were then digested by a mixture of HNO_3_ (66.66%) and HCl (33.33%) in a pressurized vacuum microwave system (Multiwave 3000; Anton Paar GmbH, Ostfildern, Germany). Finally, these samples were analyzed using ICP-AES (with the detection limit of below 100 ppb) to measure Pb and As concentrations in the plant organs as described by [65].

### 4.6. Statistical Analysis

All analyses were performed in four replicates. After verifying the data normality, One-way analysis of variance (ANOVA) was performed to estimate the significant differences (*p* < 0.05) between mean observations by using statistical software package of SPSS (SPSS, version 27.0, Inc., Chicago, IL, USA). Furthermore, R program (version 4.2.1) was used to calculate the Pearson correlation coefficient to assess the potential relation between soil properties, plant growth and metal(loid)s concentration.

## 5. Conclusions

The soil of the former mining site (Pontgibaud technosol) is characterized by high concentrations and mobility of As and Pb, which are hazardous to the environment. Rehabilitation of such an extensive polluted area must be remediated prior to the introduction of plants with the characteristics to stabilize and reduce the mobility of metal(loid). A combination of compost and biochar have been long known for their efficacy towards improving soil characteristics with a synergistic effect. However, the majority of already published studies used constant dosage of soil amendments with changed ratio. This study compared the effect of different application rates of compost and biochar to find the optimum mixture to improve the polluted soil conditions. All modalities improved soil pore water pH, electrical conductivity, stabilized Pb and increased As mobilization, but only the modality containing 20% compost with 6% biochar showed better plant growth in terms of dry weight. These results suggest that when compost and biochar are added in combination, their effects on improving plant growth may not be additive and could depend on the application rate. Moreover, the combination of compost and biochar can effectively stabilize heavy metals in the soil, which has the potential to prevent their uptake by plants and reduce their mobility in the environment. Therefore, further studies are recommended to investigate the mechanisms underlying synergistic or antagonistic interactions between compost and biochar at different application rates, and to evaluate the advantage of heavy metal stabilization in soil against phytoextraction to ensure the reliability and practical applicability of these combination of soil amendments Moreover, evaluating the effect of these combination on more suitable plants for phytoremediation purposes could provide more information on potential of the application of combination amendments in real world scenarios for future research in the field of phytoremediation.

## Figures and Tables

**Table 1:** Different soil mixtures group and treatment designations and percentage of different amendments (*w*/*w*) per treatment.

Group	Abbreviation	Soil Mixtures
Control	P100	100% Pontgibaud
1	P80C20B0	80% Pontgibaud; 20% compost
	P80C20B2	80% Pontgibaud; 20% compost; 2% biochar
	P80C20B6	80% Pontgibaud; 20% compost; 6% biochar
2	P60C40B0	60% Pontgibaud; 40% compost
	P60C40B2	60% Pontgibaud; 40% compost; 2% biochar
	P60C40B6	60% Pontgibaud; 40% compost; 6% biochar
3	P40C60B0	40% Pontgibaud; 60% compost
	P40C60B2	40% Pontgibaud; 60% compost; 2% biochar
	P40C60B6	40% Pontgibaud; 60% compost; 6% biochar

## Data Availability

The data used to support the findings of this study are available from the corresponding author upon request.

## Supplementary Materials

The following supporting information can be downloaded at: https://www.mdpi.com/article/10.3390/plants12112077/s1, Table S1: Properties of Pontgibaud technosol, biochar and compost used in the experiment.
