# Supplementary material for: Importance of Application Rates of Compost and Biochar on Soil Metal(Loid) Immobilization and Plant Growth
Source: Plants (Basel). 2023 May 23;12(11):2077. doi: 10.3390/plants12112077 (PMC10255519; doi:10.3390/plants12112077)
Supplement: Supplementary file 1 [file plants-12-02077-s001.zip › plants-2382135-supplementary.pdf]

Table S1: Properties of Pontgibaud technosol, biochar and compost used in the experiment.

	Properties	Value	Reference
Pontgibaud technosol	pH	4.80 - 4.99	29 - 31
	EC (µS.cm^{-1})	262 - 599	
	[As] (mg.L^{-1})	0.01 - 0.022	
	[Pb] (mg.L^{-1})	12.307 - 56.97	
	Organic matter content (%)	1.42	
	Total nitrogen (mg.kg^{-1})	75	
	Cation exchange capacity (% CaCO3)	0.7	
Biochar	Particle size (mm)	0.5–1	32
	pH	8.46	
	EC (µS.cm^{-1})	302	
	WHC (%)	212	
	Specific area (m2.g^{-1})	4.38	
	Total pore volume (cm3.g^{-1})	0.01	
	Mean pore diameter (nm)	9.13	
Compost	Particle size (mm)	0-5	Klasmann-Deilmann, Saint-Louis-du-Rhône: France
	pH	6	
	EC (mS.cm^{-1})	35	
	Water retention capacity	80%	
